# Computational single fundus image restoration techniques: a review

**DOI:** 10.3389/fopht.2024.1332197

**Published:** 2024-06-12

**Authors:** Shuhe Zhang, Carroll A. B. Webers, Tos T. J. M. Berendschot

**Affiliations:** University Eye Clinic Maastricht, Maastricht University Medical Center, Maastricht, Netherlands

**Keywords:** retinal image, image enhancement, image restoration, illumination correction, dehazing, deblurring, diagnosis

## Abstract

Fundus cameras are widely used by ophthalmologists for monitoring and diagnosing retinal pathologies. Unfortunately, no optical system is perfect, and the visibility of retinal images can be greatly degraded due to the presence of problematic illumination, intraocular scattering, or blurriness caused by sudden movements. To improve image quality, different retinal image restoration/enhancement techniques have been developed, which play an important role in improving the performance of various clinical and computer-assisted applications. This paper gives a comprehensive review of these restoration/enhancement techniques, discusses their underlying mathematical models, and shows how they may be effectively applied in real-life practice to increase the visual quality of retinal images for potential clinical applications including diagnosis and retinal structure recognition. All three main topics of retinal image restoration/enhancement techniques, i.e., illumination correction, dehazing, and deblurring, are addressed. Finally, some considerations about challenges and the future scope of retinal image restoration/enhancement techniques will be discussed.

## 1 Introduction

**Figure 1:**
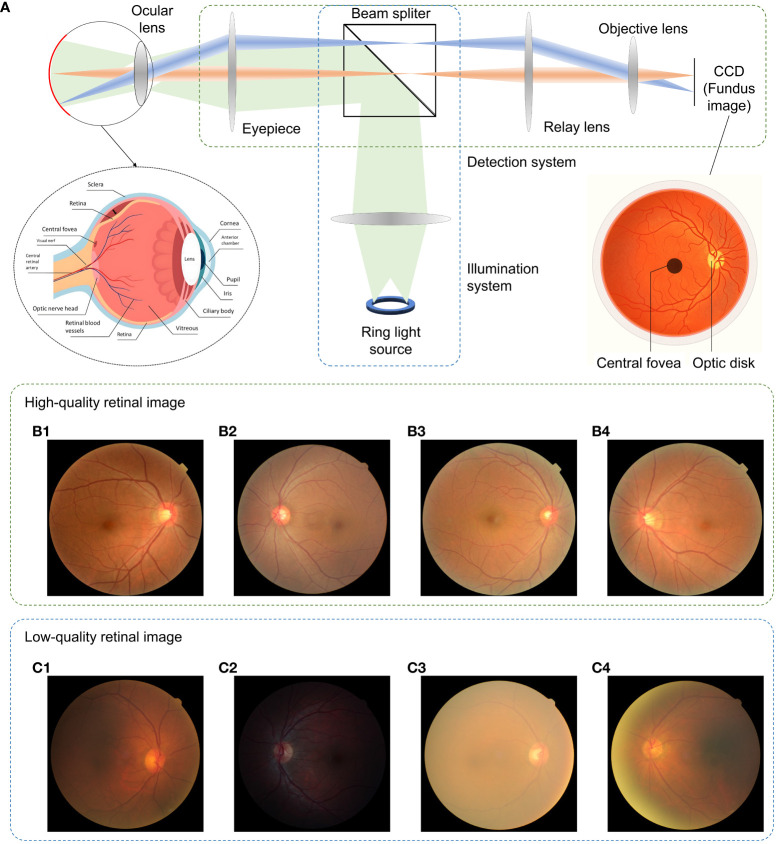
Fundus camera and demonstration of retinal images of good and low quality. **(A)** Sketch of optical design of a fundus camera. **(B1–B4)** are sample high-quality images. **(C1)** Low-quality retinal image with haze and uneven illumination. **(C2)** Insufficient illumination. **(C3)** Haze effect. **(C4)** Uneven illumination and blurriness.

The introduction of the ophthalmoscope by Helmholtz (1) allowed one to obtain images of the retina and put ophthalmology on the map as a separate sub-area of medicine. In his design, the ophthalmoscope, the subject’s eye, and the examiner’s eye together form two optical systems that become the classical design of the successor of the fundus camera where the examiner’s eye is replaced by a camera sensor. A typical retinal imaging platform thus can be regarded as two coupled imaging systems, as shown in **Figure 1A** (2, 3). One is the ocular system, and the other is a reflective imaging system that normally illuminates the fundus through the pupil and collects the reflected light from the retina, forming the image on the camera sensor. Ophthalmologists have been using retinal images for the early detection, diagnosis, and monitoring of ocular diseases and their progression. Morphologic changes due to eye diseases like diabetic retinopathy (4–7), solar retinopathy (8), glaucoma (9–11), and age-related macular degeneration (12–14) can be directly observed in these images. In addition, neurological diseases such as stroke and cognitive dysfunction can also be diagnosed through retinal images (15, 16) as well as cardiovascular risk factors (17–19). Diagnosing efficiency and precision are deeply related to the quality of retinal imaging. Obviously, the higher the image clarity, the more detailed information can be observed from the image, and the better their diagnostic capabilities. **Figures 1B1**–**B4** shows the fundus images with good visual quality. However, not every retinal image is perfect and low-quality image occurrence is not a minor problem. Heaven et al. found 9.5% of all acquired images to be entirely unsatisfactory in a prospective study of 981 patients with diabetic retinopathy (20). Scanlon and Stephen found the ungradable image rate to be between 19.7% for nonmydriatic photography and 3.7% for the mydriatic photography study of 3,650 patients with diabetes (21).

Retinal images can be severely degraded by opacities in the optical media of cataract eyes (22–24), as shown in **Figures 1C1–C4**, and retinal images for non-cataract subjects can be degraded by poor illumination conditions including uneven or insufficient illuminations. The quality of retinal imaging can be improved by using high-end fundus cameras such as adaptive optics and laser-based fundus cameras to tackle the optical aberrations (25) and media opacities, but will increase financial pressures and limit access to healthcare for patients since not all clinics have such high-end equipment. In contrast, image enhancement processing offers affordable and efficient solution to digitally increase the image’s quality, such as to correct for illumination artifacts (26–28), to enhance contrast (29–31), and to reveal the effect of dehazing algorithms on cataract (32–34).

To increase the image clarity again, several contrast-enhancement methods have been proposed, which can be divided into two major categories: data domain methods and restored model methods (35). Data domain methods are further divided into two types based on their algorithms. The first type is known as the transform-domain algorithm, which transforms a raw image into a new function of other parameters such as the spatio-frequency domain corresponding to Fourier transform (36) or the structure feature domain corresponding to top-hat transform (37, 38). The image is processed in the transformed domain and then transformed back, resulting in a new image with enhanced contrast. The transform-domain algorithm enables us to globally or locally modify the weight for different structures within the image. However, owing to computation costs, the image-domain algorithm, which is the second type of data domain methods, is favored.

The core idea of the image-domain algorithm is the gray-level adjustment. Histogram equalization (HE) and its improved version, contrast-limited adaptive histogram equalization (CLAHE), are usually used for quick retinal image enhancement (29). Other histogram modification methods such as q-quantile (39) and the gray-scale global spatial entropy method also show promising results in improving the image’s contrast (10, 40). Global gray-level adjustment methods including the gamma map (41) and *α*-rooting (42) use a fixed function to convert the global gray-level distribution for adjusting the brightness of retina imaging.

Another group of image-domain algorithms uses filters to enhance contrast. These algorithms are similar to transform-domain algorithms but use a convolution kernel to separate the background and foreground information of an image (42, 43). The foreground information usually corresponds to the detailed structure of an image. By modifying the weights between background and foreground, the contrast of the detailed structure can be enhanced. In general, data domain methods belong to pure signal (image) processing techniques that normally take a few considerations of the physical insight of the image formation and enhancement.

In order to obtain self-consistent methods for retinal imaging enhancement, restored model methods have been developed as they digitally inverse the progress of how a degraded image was formed. These restored model methods share a similar idea of computational imaging, i.e., a physical model describes the optical process of forming an image under the impact of degeneration agents, which could be optical aberrations, unstable vibrations, or limited optical resolution. By directly or indirectly measuring the optical properties of these degeneration agents, one can compensate for the degeneration agents by digitally mimicking the propagation of the optical wave and modifying the wavefront of light (44–47). Imaging through scattering media, for example, is a well-known application of computational imaging (46, 47).

Different from computational imaging, the restored models for imaging enhancement do not measure the optical properties of the degeneration agents but try to find solutions corresponding to statistical properties in optical or visual aspects. The solutions can be regarded as rough estimated versions of those degeneration agents and can be also compensated by applying them to the image formation model, resulting in enhanced images.

Restored model methods are widely used for image dehazing (48, 49), underwater image enhancement, and night image enhancement (50); however, only a few studies have reported their use in retinal imaging enhancement. To our knowledge, the first publication about the application of restored model methods in retinal image enhancement can be traced back to 1989 (22), where the model for imaging the retina in photographs taken through intraocular scatter is considered similar to the model used to represent imaging of the earth from a satellite in the presence of light cloud cover. Here, scattering was removed (or suppressed) by using the Retinex theory.

Based on an image formation model, Xiong et al. (35) used intensity correction and histogram adjustment to preprocess the image, after which a transmission map was generated according to the intensity of the preprocessed image in each color channel. Haze could be suppressed through dehazing. Although the performance of their approach is good, it relies on statistical and empirical properties of the particular retina imaging database to determine the algorithm parameters, which makes it hard to apply in different databases. A subsequent study (26) used the illumination-reflectance model of image formation to correct the illumination of retinal images; the research shows that illumination correction is mathematically equivalent to dehazing when the color of the image is reversed. With that, the color-reversed dark-channel prior (DCP), also known as bright-channel prior (51), was employed, showing an efficient illumination correction. Following the haze image formation model and dehazing, Mitra et al. (52) proposed a “thin layer of cataracts” model to achieve contrast enhancement for cataract fundus images.

**Figure 2:**
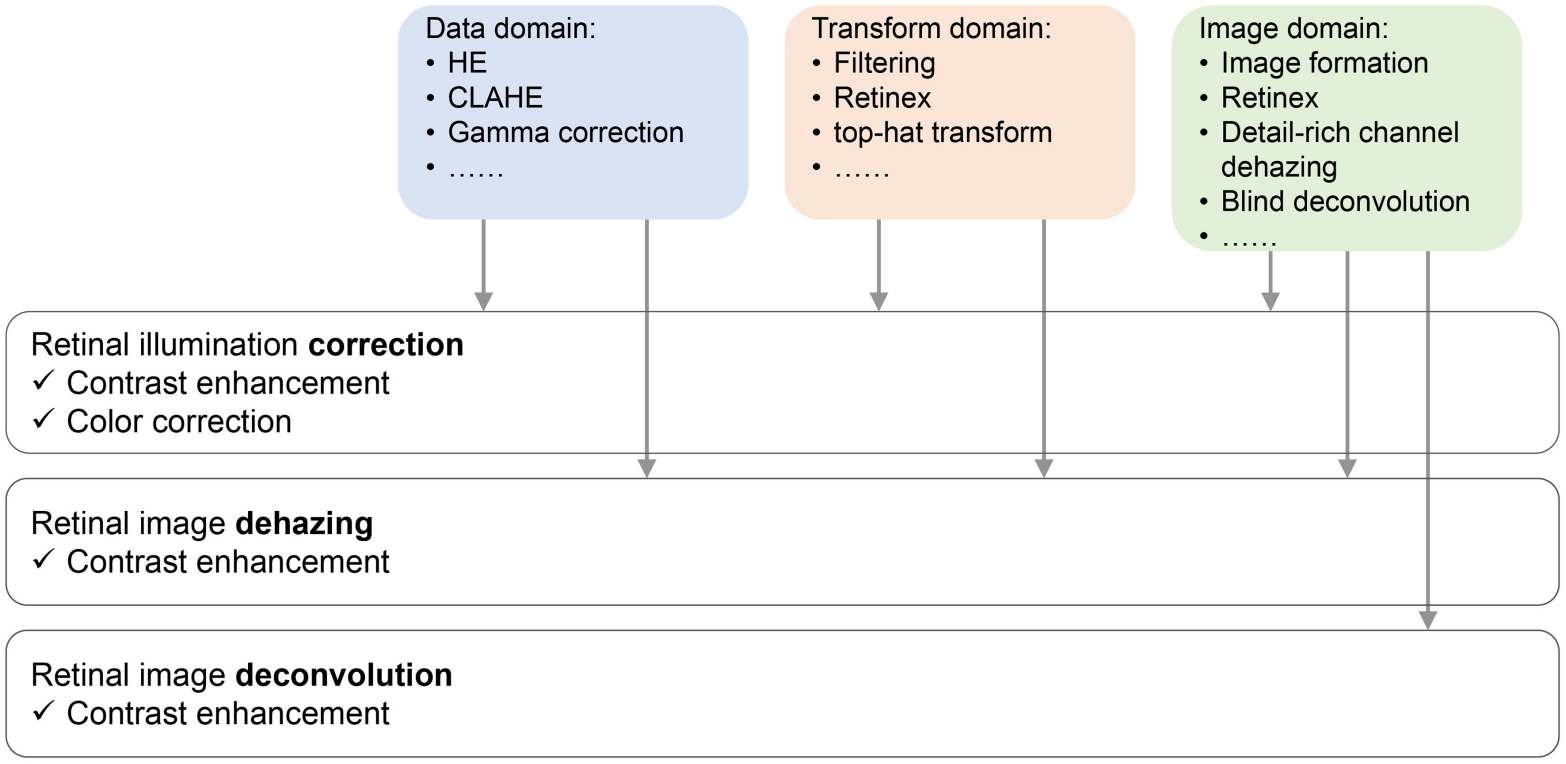
Sketch of retinal image restoration tasks, their solutions, and the effect they bring to the enhanced images.

Gaudio et al. (53) demonstrated a pixel color amplification method for retina imaging enhancement, which shows good performance in enhancing the detailed structure of retina images. **Figure 2** plots the categories of fundus retinal image enhancement algorithm, and their applications.

In this review paper, we first revisit the mathematical model used for retinal image restoration, their physical/mathematical insight, and how they are related to each other in Section 2. We further show how these image formation models are applied to retinal image restoration in illumination correction, dehazing, and deblurring in Section 3. A brief introduction to deep-learning-based methods is also discussed. In Section 4, the significance and benefits of the clinical applications of retinal image restoration techniques are discussed, and the social impact of retinal image enhancement is described in Section 5. The concluding remarks and future scope are presented in Section 6.

## 2 Mathematical models for retinal image restoration

### 2.1 Pixel value stretch model

Enhancing the quality of the retinal image can be achieved by manipulating pixel values. For example, one can enlarge pixel values if the original values are too small to be noticed or decrease them if they are too bright.

Accordingly, the gamma correction, given in Equation (1), provides a straightforward way for pixel adjusting, which is widely used in medical image analysis (54).


(1) $$s_{\text{o}\text{u}\text{t}\text{p}\text{u}\text{t}}=s_{\text{i}\text{n}\text{p}\text{u}\text{t}}^{\gamma }$$

where $s_{\text{o}\text{l}\text{d}}$ is the input image and $s_{\text{n}\text{e}\text{w}}$ denotes the output image after the gamma correction. When γ*<* 1, the nonlinear transforms the small value of pixels to large value so that the pixels become bright, while if γ *>* 1, the small value is further suppressed.

**Figure 3:**
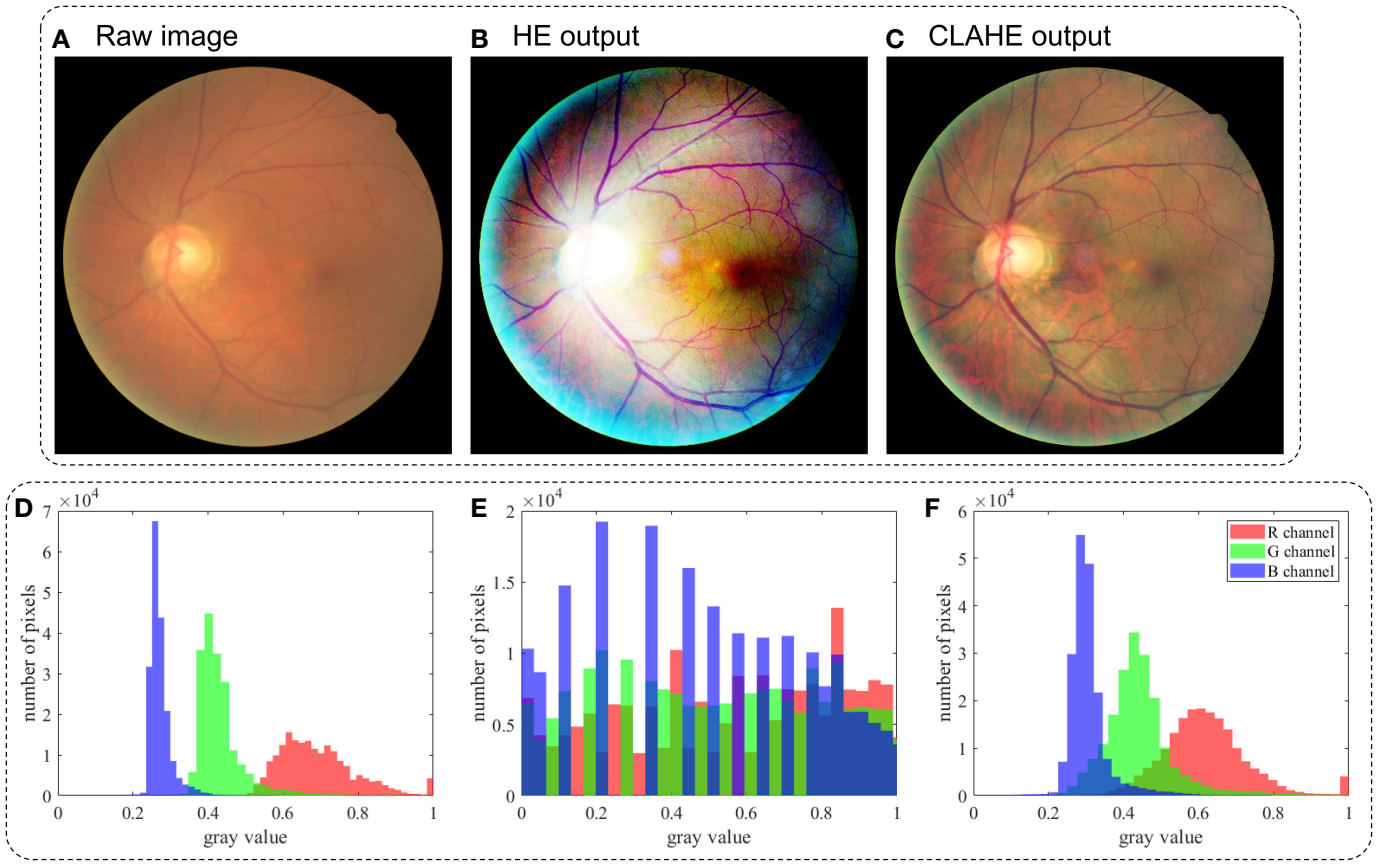
Sketch of retinal image restoration tasks and their solutions. **(A)** Raw image. **(B)** Output image enhanced by HE. **(C)** Output image enhanced by CLAHE **(D)**, **(E)** and **(F)** Histogram of pixel value for image in **(A)**, **(B)** and **(C)**, respectively.

Another method for pixel adjustment is HE. Taking the image in **Figure 3A** as example, when the image is represented by a narrow range of intensity values, HE is able to make the intensity better distributed among the full dynamic range, as shown in **Figures 3B, F**. To avoid the over- and underexposure effect of HE, CLAHE (55) is proposed to adaptively achieve HE according to the local contrast in the image’s sub-block.

HE and its improved version, CLAHE, are widely used as preprocessing methods for retinal image enhancement, and the research has shown that the image formation model-based methods gain better image restoration results than HE methods as shown in **Figure 3C** and **F**.

### 2.2 Image formation model

**Figure 4:**
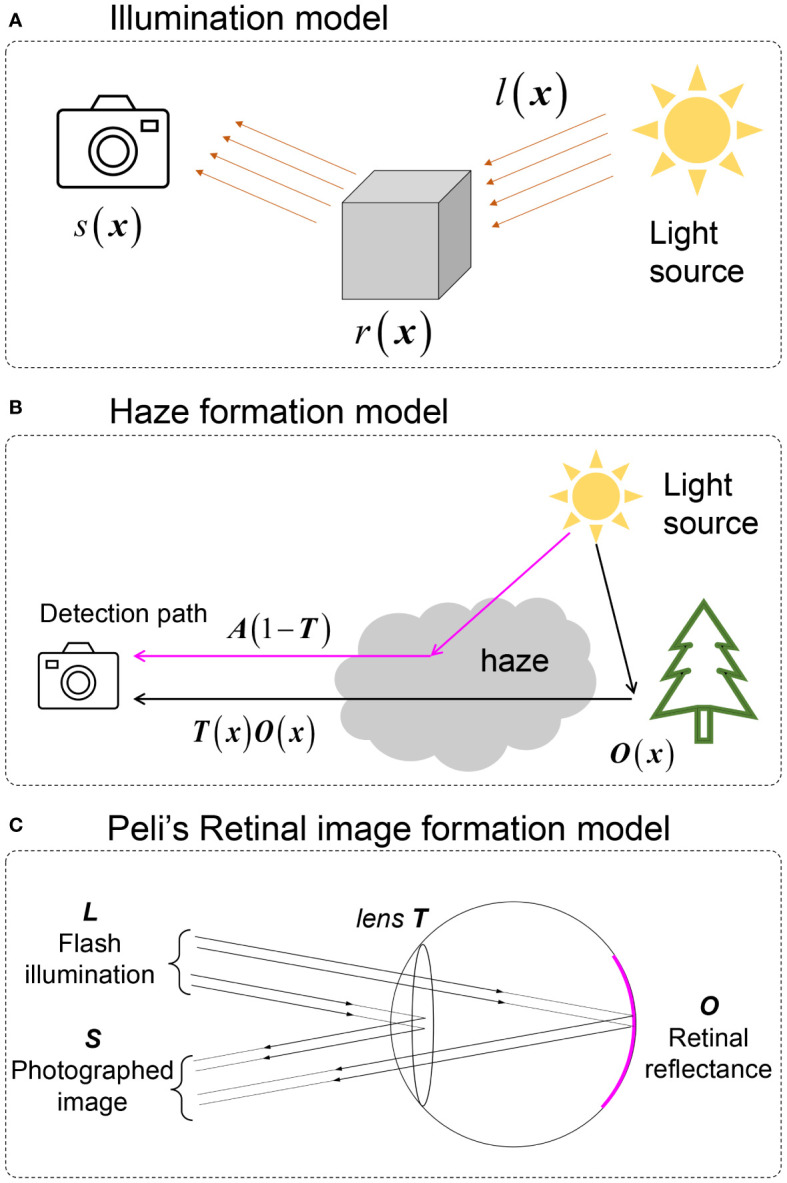
Image formation models involved in retinal image enhancement. **(A)** illumination model. **(B)** Natural scene haze formation model. **(C)** Peli’s retinal image formation model.


A widely used image formation model for retinal image enhancement is the illumination model, shown in **Figure 4A**, given by

(2) s(x) =l(x) ·r(x).

Here, · denotes elementwise multiplication, **x** denotes the spatial coordinates, *s* is the captured image by the camera, *l* is the illumination pattern from the light source that is assumed to be spatially slow varying, and *r* is the retinal reflectance.

To tackle the haze effect caused by intraocular scattering on retinal imaging, a haze formation model is adopted. The early-stage model was directly adopted from Koschmieder and McCartney’s model (56, 57) of hazy nature scenes shown in **Figure 4B** and, given by


(3) s(x) =t(x) ·o(x) +a[1 −t(x)].

Here, *o*(**x**) is the haze-free image, and *t*(**x**) is the transmission matrix of the haze medium describing the portion of the light that is not scattered and reaches the camera. *a* is the global atmospheric light, and *s*(**x**) is the observed image. Despite the fact that a large number of natural scene dehazing studies were based on Equation (4) (58), this was developed for natural scenes and is not the optimal choice for fundus imaging since it ignores the double-pass property of fundus photography.

To establish an image formation model for retinal imaging, Peli et al. (22) developed an optical model for imaging the retina through cataracts, which is


(4) s(x) =α·l·o(x) +l[1 −t(x)].

where *l* is considered to be the flash illumination of the fundus camera and *α* is the attenuation of retinal illumination due to the cataract. Both *l* and *α* are considered to be constant. Different from Equations (3)-(4), reveals that the illumination pattern also impacts the quality of retinal imaging. However, as *l* is constant, Equation (4) loses the ability to correct the uneven (spatially varying) illumination of retinal imaging. In addition, the existing parameter *α* shows the idea of the double-pass property where the illumination light interacts twice with the cataract layer (when the light goes inside the eye and when it is reflected out from the fundus).

**Figure 5:**
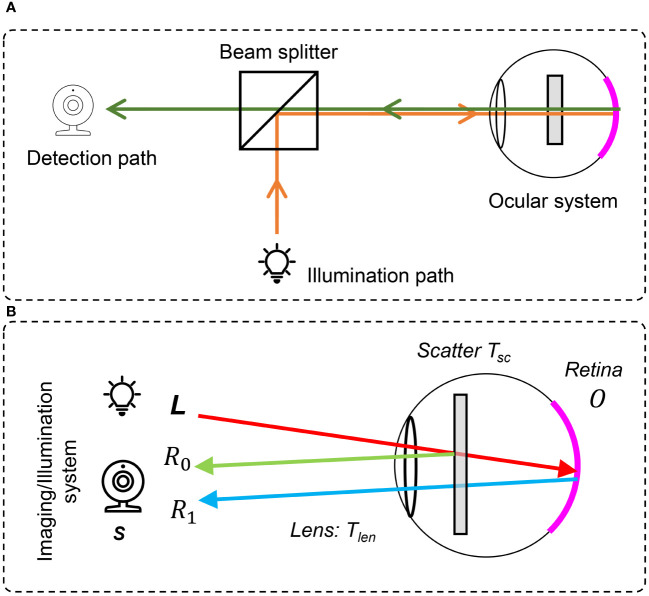
The double-pass fundus reflection model. **(A)** Optical path in a fundus camera. **(B)** Sketch of double-pass fundus reflection.

In a previous study (33), we proposed the double-pass fundus reflection (DPFR) that deals with image formation in retinal imaging as shown in **Figure 5A** and **B**. This DPFR model is given by


(5) $$s\left(x\right)=l\left(x\right)\cdot \left[t^{2}\left(x\right)\cdot o\left(x\right)+1-t\left(x\right)\right],$$

where *l*(**x**) is the illumination from the outside of the eye and is delivered by the illumination system of the fundus camera. Different from the nature scenes’ hazy formula in Equation (3), the transmission matrix *t* is squared, denoting the double-pass feature of fundus imaging (59, 60), where incident light will transmit twice through the pupil.

We ignore the illumination light color of a fundus camera and assume that the retina is illuminated by ideal white light (identical value in R, G, and B channels), which may have an uneven and insufficient illumination pattern. *t*(**x**) is the transmission matrix of intraocular scatter including ocular lens and cataract layers. Equation (5) reveals that the degeneration of the retinal image is mainly due to three parts (1): an uneven illumination condition (2), filtering by the human lens, and (3) intraocular scattering.

### 2.3 Image structures model

**Figure 6:**
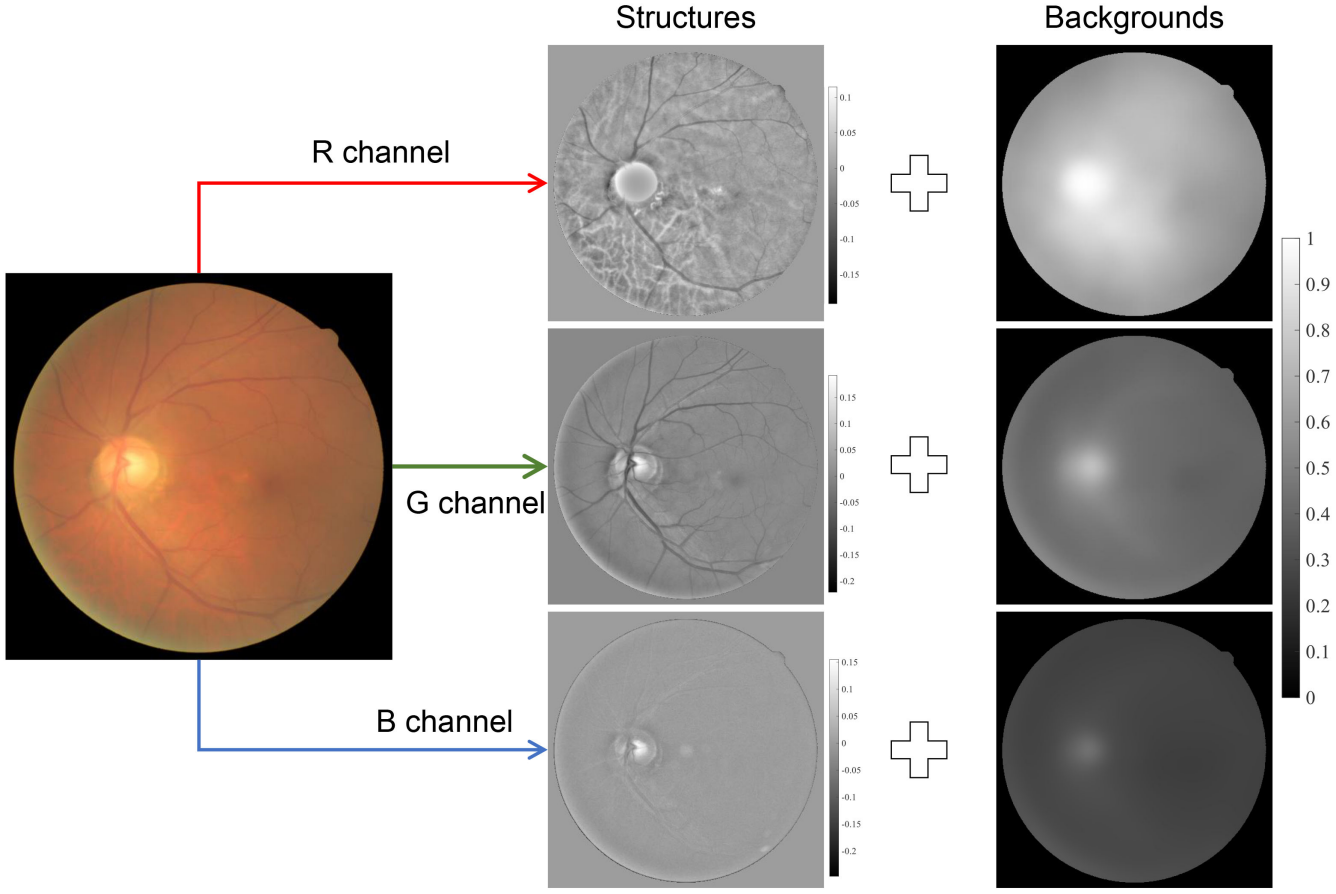
Image structure decomposition.

Besides the image formation model, there are also image structure models used for retinal image enhancement (31, 42, 43, 61), and they can be summarized as


(6) $$s\left(x\right)=s_{\text{background}}\left(x\right)+s_{\text{structures}}\left(x\right).$$

where $s_{\text{background}}$ is the background information of the observed image that corresponds to the low-frequency components, while $s_{\text{structures}}$ denotes the detailed information implying the detailed structures and textures of the image, as shown in **Figure 6**. By giving a large weight to $s_{\text{structures}}$ and suppressing the $s_{\text{background}}$ , one can obtain a contrast-enhanced image. The background components, $s_{\text{background}}$ , can be obtained by low-pass filtering of *s*(**x**) (42, 61) and total variation regularization (31), while $s_{\text{structures}}$ can be obtained by high-pass filtering of *s*(**x**) or subtracting $s_{\text{background}}$ from *s*(**x**). Note that Equation (6) is not based on the optical process of how the image is formed and the physical insight is different from Equations (2) and (4).

### 2.4 Retinex theory

It is worth noting that the illumination model [Equation (2)] and image structure model [Equation (6)] can be unified by the Retinex theory, which was developed first to explain the land effect in a visual prospective (62). It was later developed for uneven illumination correction in computer vision. Retinex can be categorized into several types including variational Retinex (63–65), PDE Retinex (66–68), threshold Retinex (69), and center/surrounded Retinex (known also as filtering-based Retinex) (70, 71), while the filtering-based Retinex gained a lot of research interest due to its computational efficiency and simple implementation. Taking the logarithm to both sides of Equation (2), we obtain


(7) logs(x)=logl(x)+logr(x),

which is identical to Equation (6) in their mathematical forms, so that the illumination component is split as a linear term that is added to the reflection component. Since *l*(**x**) is assumed to be spatially slow-varying, a good estimation of *l*(**x**) can be given by low-pass filtering of *s*(**x**). Then, the reflectance *r*(**x**) is given as


(8) r=exp{logs−log(F⊗s)},

where *F* is a low-passing filter, which is known also as surround function. ⊗ denotes the 2D convolution. In practical implementation, a pixel value normalization should be applied to Equation (8), to avoid distorting pixel intensity.

## 3 Retinal image restoration

### 3.1 Intensity correction

Retinal image intensity correction is a very important task for retinal image restoration. Statistical analysis shows that many retinal images suffer from problematic, uneven, and insufficient illumination, which is highly related to the performance of photographers, the imperfect head/eye position of subject participants, and the potential poorly designed illumination path of the fundus camera.

Since human visual assessment on image quality is highly related to the image’s brightness, intensity correction on a retinal image can produce significant improvements on the image’s quality for visual assessment. In this section, we briefly introduce two solutions according to Section 2—the gamma correction and Retinex method for retinal image intensity corrections—and demonstrate their output on sample fundus images.

#### 3.1.1 Gamma correction

**Figure 7:**
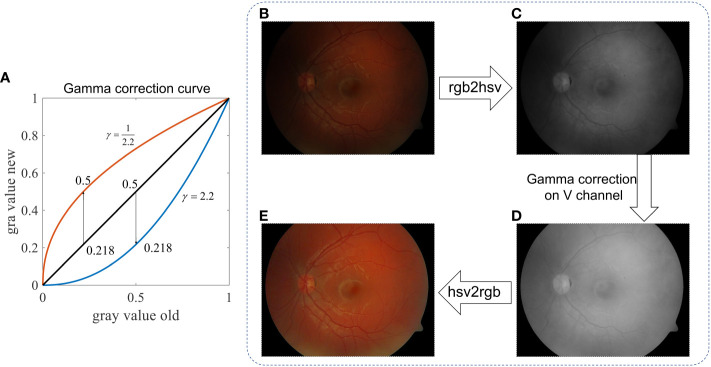
Retinal image intensity correction using Gamma correction. **(A)** Gray value curve shows the mapping of gamma correction. **(B)** Raw image. **(C)** V-channel of image in the HSV color space. **(D)** V-channel after Gamma correction. **(E)** Enhanced image.

An intensity correction can be achieved by a Gamma correction if γ *<* 1 in Equation (1). As shown in **Figure 7A**, when γ = ½.2, a small value, say, 0.218, becomes 0.5 after the Gamma correction. Accordingly, we can transform the input RGB retinal image shown in **Figure 7B** to the HSV-color space, and then perform a gamma correction to its V-channel (Value). After that, the image is transformed back to the RGB-color space, resulting in illumination-corrected images, as shown in **Figure 7E**. By adjusting the value of γ, one can achieve different strengths of illumination correction, while the image contrast is not yet significantly improved.

#### 3.1.2 Center-surrounded Retinex

**Figure 8:**
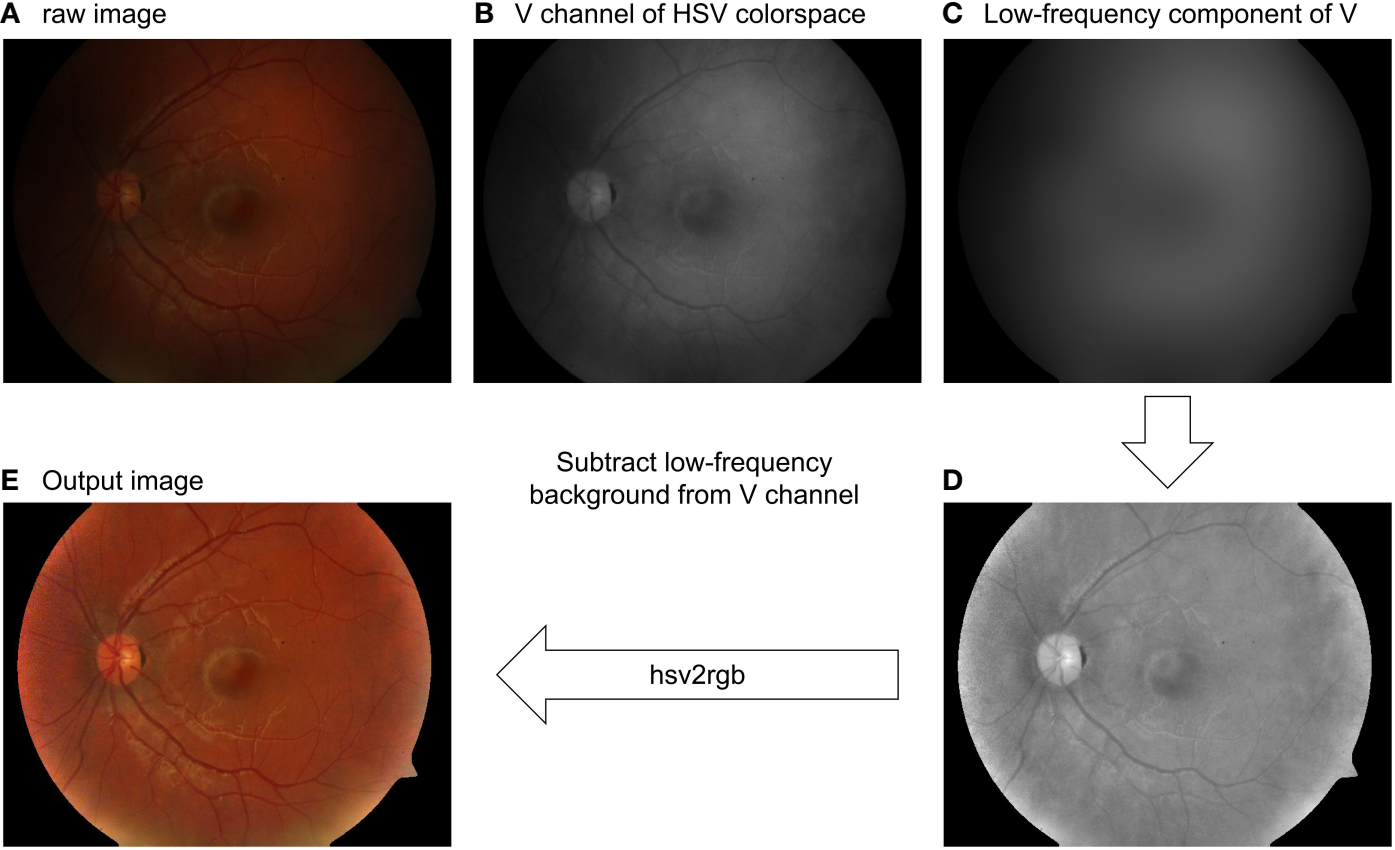
Retinal image intensity correction using the Gaussian filtering Retinex method. **(A)** Raw image. **(B)** V-channel of the raw image. **(C)** Low-pass filtering of **(B)**. **(D)** Intensity-corrected V-channel. **(E)** Output image.

As mentioned in Section 2.4, intensity correction can also be achieved using Retinex. The low-frequency component of the V-channel can be a good estimation of the illumination pattern, as shown in **Figure 8A, B** and **C**. Subtracting the illumination pattern from the original V-channel (**Figure 8D**) and applying the intensity normalization so as the intensity value is between 0 and 1, the output V-channel is shown in **Figures 8D, E** for the RGB image where the uneven pattern is corrected.

Besides the Gaussian-filtering Retinex method shown above, researchers have developed a more complex framework, such as variational Retinex (63–65) with a different regularization and non-local Retinex (72) to achieve illumination correction. The application on the retinal image and how the retinal image can benefit from the Retinex method can attract a large amount of research interest. It is also worth noting that the Retinex theory linked image illumination correction and image dehazing through simple algebra. This property will be further discussed in Section 3.2.2.

### 3.2 Dehazing

In case of intraocular scattering, the captured image may have a haze-like effect, which is similar to the haze effect occurring in natural scenes. In these cases, a dehazing process is needed to enhance the quality of retinal images.

#### 3.2.1 Dehazing using dark-channel prior

The DCP (73) has been widely used for natural scene dehazing including underwater image enhancement and haze removal even for thick fog situations. Here, the dark channel is obtained by first filtering the three color channels of the image using a local minimum filter with a size of w pixels, and then calculating the minimum value within three color channels.

**Figure 9:**
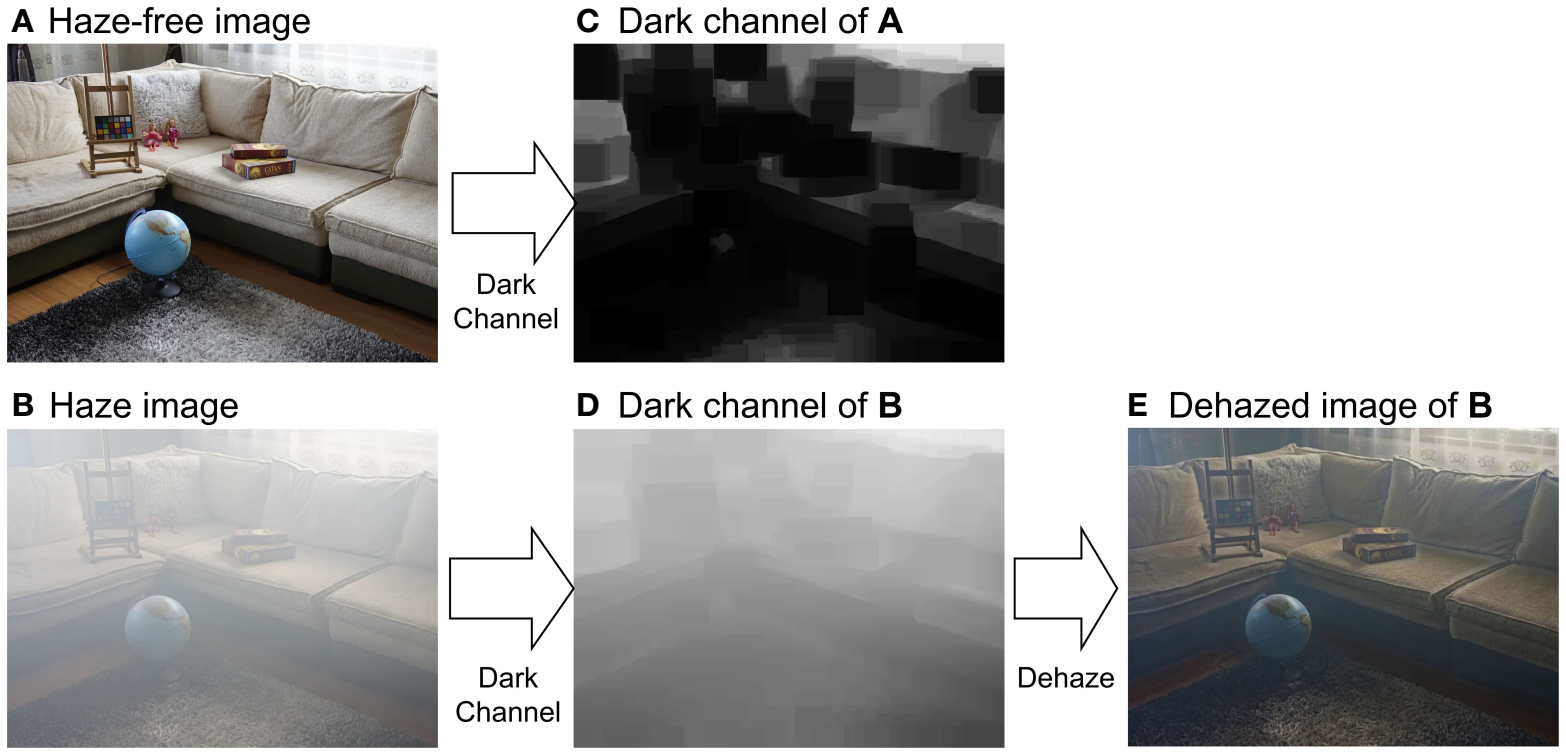
Dehazing using dark-channel prior. **(A)** Hazy-free image. **(B)** Haze image of **(A)**. **(C, D)** are dark channels of **(A, B)**, respectively. **(C)** is the dehazed image. **(E)** is the dehazed image.

The principle of DCP tells that in any haze-free image (in RGB color space), as shown in **Figure 9A**, at least one pixel has zero intensity in at least one channel, as shown in **Figure 9C**. As such, the transmission map of a hazy image (**Figure 9B**) can be estimated using the dark channel of the image, as shown in **Figure 9D**. According to the image formation model in Equation (3), the dehazed image can then be calculated.

Although results of DCP dehazing are promising, the performance of DCP on retinal image dehazing is limited, especially for thick cataracts due to different color statistical features between natural scene images and retinal images. DPC fails to estimate the transmission map of the retinal image in RGB color space; however, it works in the intensity domain since DCP is valid for gray-scaled image dehazing (74).

**Figure 10:**
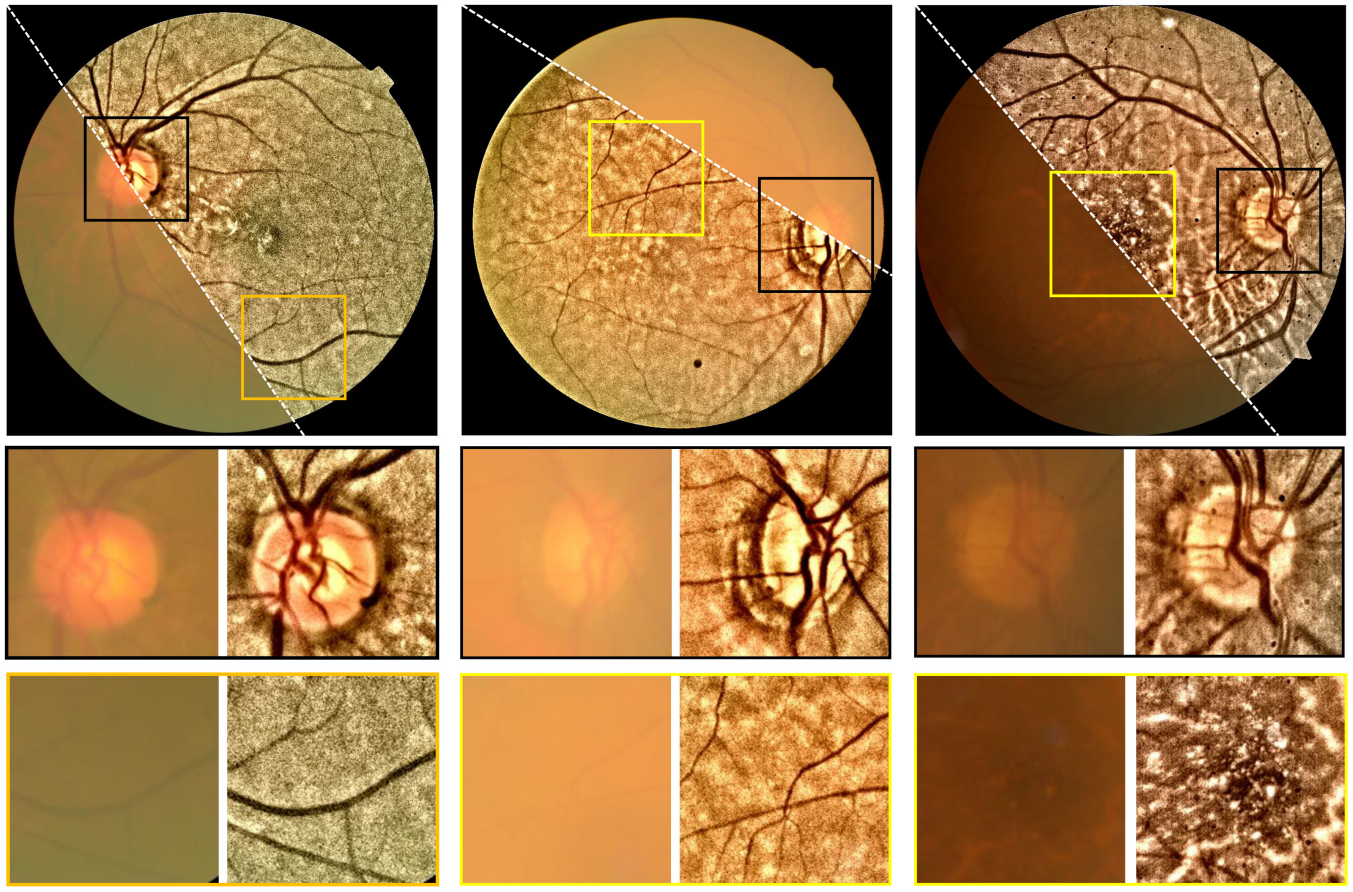
Restoration of cataractous retinal images. First row: raw images. Second row: restored images. The image was dehazed using the DCP after the illumination correction was performed.

Accordingly, one is able to convert the retinal image from RGB color space to, for example, the CIE-LAB color space, and then perform dehazing to the L-channel (intensity channel). After that, the dehazed retinal image is obtained. **Figure 10** shows the dehazing results on the cataractous retinal image after the illumination correction was applied. The haze effect is significantly suppressed.

#### 3.2.2 The duality between intensity correction and dehazing

**Figure 11:**
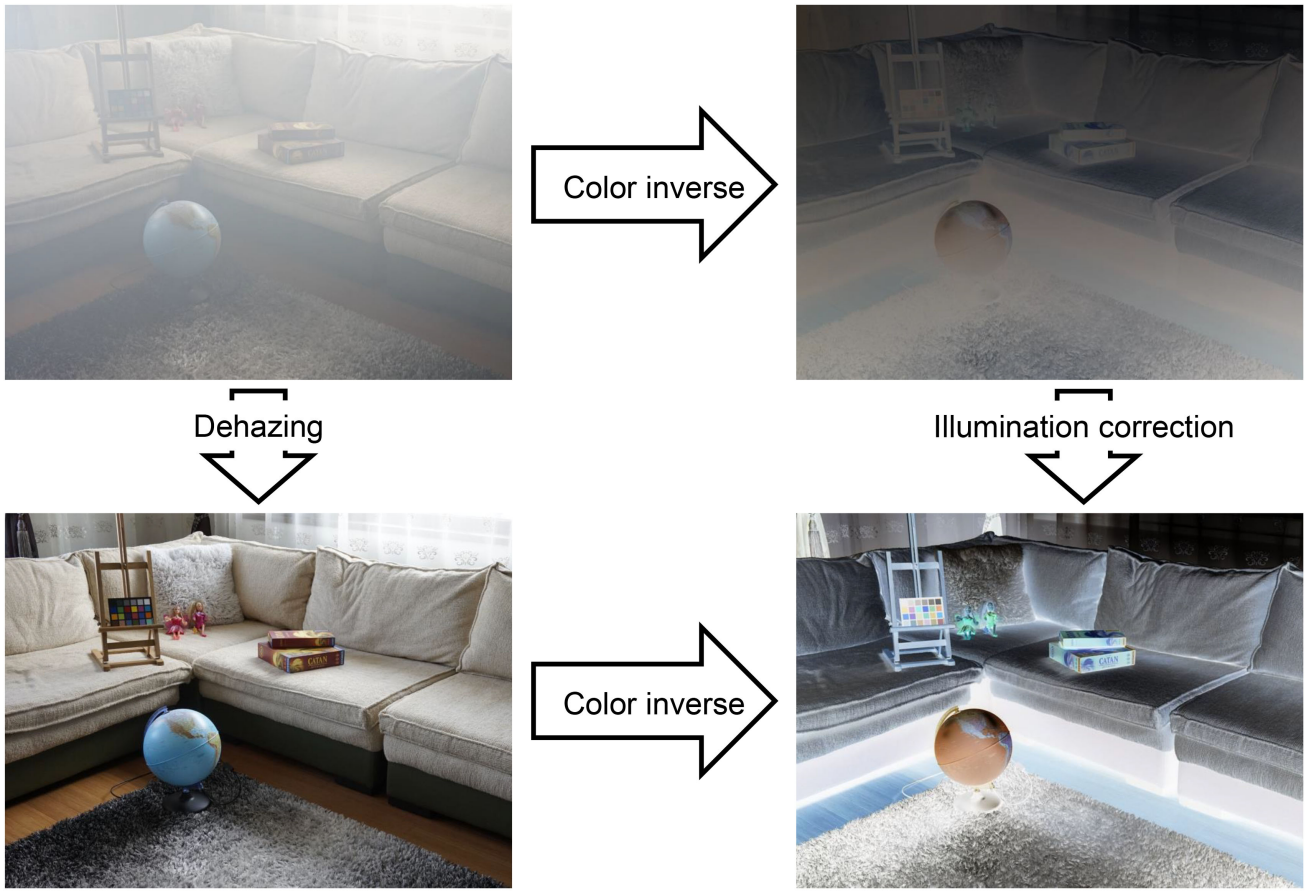
Dehazing task can be converted into an intensity correction task in color-inversed domain.

Nature scene image dehazing seems to be unrelated to intensity correction since they deal with different problems. Later, as pointed out in Ref (69)., they are connected by an algebra modification of the haze formation model in Equation (3) by assuming that the input image is globally white-balanced, that is, s=t·o+ (1 −t) . With some algebra, it can be rewritten as (1 −s) =t· (1 −o) . By considering $\left(1\;-s\right)\;=s_{\text{n}\text{e}\text{w}}$ and (1 −o) =r , we are able to convert the haze formation model to the illumination model in Equation (2). This implies an interesting phenomenon such that the color-inversed hazy image looks like an image suffering from insufficient illumination, as shown in **Figure 11**.

According to the Retinex theory, by assuming *t* is spatially slow-varying and using Equations (2) and Equation (3), we have


(9) o=1−Retinex(1−s)=Dehazing(s).

It is also proven in (75), and shows that the dehazing task can be finished under the Retinex theory. Acccording to Equation (9), the Retinex theory is the bridge to image dehazing and image illumination correction (76). The application of the Retinex theory in retinal image dehazing shares a similar idea of an image structure model and filtering-based Retinex, where the haze layer is regarded as the slow-varying background component of the retinal image, and the dehazed image can be obtained by subtracting the background component from the hazy one (42, 61).

Generally speaking, algorithms for retinal image illumination correction and dehazing do vary in their definition, implementation, underlying structure, and relationship that a concise description is needed to decide which to use. **Table 1** lists some of the start-of-the-art publications on non-deep-learning methods of single retinal image enhancement.

**Table 1:** List of publications on non-deep-learning based methods of single retinal image enhancement.

Literatures	Image formation model	Key idea		Functions	
			Illumination correction	Contrast enhancement	Dehazing
(22)	Equtaion (4).	Filtering, Retinex	N/A	Yes	Yes
(27)		Image Filtering	Yes	Yes	N/A
(29)		CLAHE	N/A	Yes	N/A
(43)	Equation (7).	Filtering Retinex	Yes	Yes	N/A
(77)	Equations (2). and (7)	Filtering Retinex	Yes	Yes	N/A
(26)	Equations (2). and (3)	DCP, Retinex	Yes	N/A	N/A
(35)	Equation (3).	HE, Filtering	Yes	Yes	Yes
(78)	Equation (7).	CLAHE	Yes	Yes	N/A
(79)		HE	Yes	Yes	N/A
(52)	Equation (7).	Filtering Retinex, HE	Yes	Yes	Yes
(41)	Equation (2).	Gamma correction, CLAHE	Yes	Yes	N/A
(39)	Equation (2).	Gamma correction, HE	Yes	Yes	N/A
(80)	Equation (2).	HE	Yes	Yes	N/A
(61)	Equations (2). and (7)	Filtering, Retinex	Yes	Yes	Yes
(42)	Equations (2). and (7)	Filtering, Retinex	Yes	Yes	Yes
(53)	Equation (3).	DCP, extension of DCP	Yes	Yes	Yes
(30)	Equation (3).	Extension of DCP	Yes	Yes	Yes
(31)	Equations (2). and (7)	Filtering, Retinex	Yes	Yes	N/A
(81)		Gamma correction, HE	Yes	Yes	N/A
(33)	Equation (5).	Filtering Retinex, DCP.	Yes	Yes	Yes
(82)	Equations (2). and (7)	Filtering, Retinex	Yes	Yes	N/A

### 3.3 Deblurring

Image blind deconvolution has been developed and is mainly used for natural scene image deburring (83, 84). Much prior knowledge, including but not limited to the heavy-tail prior (85, 86), gradient L0 prior (87), the dark-channel prior (88), and the local maximum gradient prior (89), has been explored to facilitate single-image blind deconvolution tasks. Nevertheless, blind deconvolution for retinal images is still problematic and challenging since there are a large number of retinal images suffering from poor illumination conditions that hide the structure (edge) information that is essential for proper deconvolutions. To the best of our knowledge, only few studies have reported on single retinal image blind deconvolution (90–93), which rather aimed to correct blurriness caused by aberrations and motions during image capture.

Andrés et al. proposed a two-step retinal image blind deconvolution method (91), in which the first step is estimating and compensating for the uneven illumination using a fourth-order polynomial. The second step is blind deconvolution with TV regularization corresponding to the heavy-tail prior to natural scene deburring. However, this method requires at least two paired retinal images of one identical subject. Francisco et al. limit the shape of the convolution kernel to a Gaussian shape and perform a line search to determine the size of the Gaussian kernel corresponding to the peak image quality score (92). This method does not correct the illumination pattern of the retinal image; in addition, not all retinal images are degraded by a simple Gaussian kernel.

**Figure 12:**
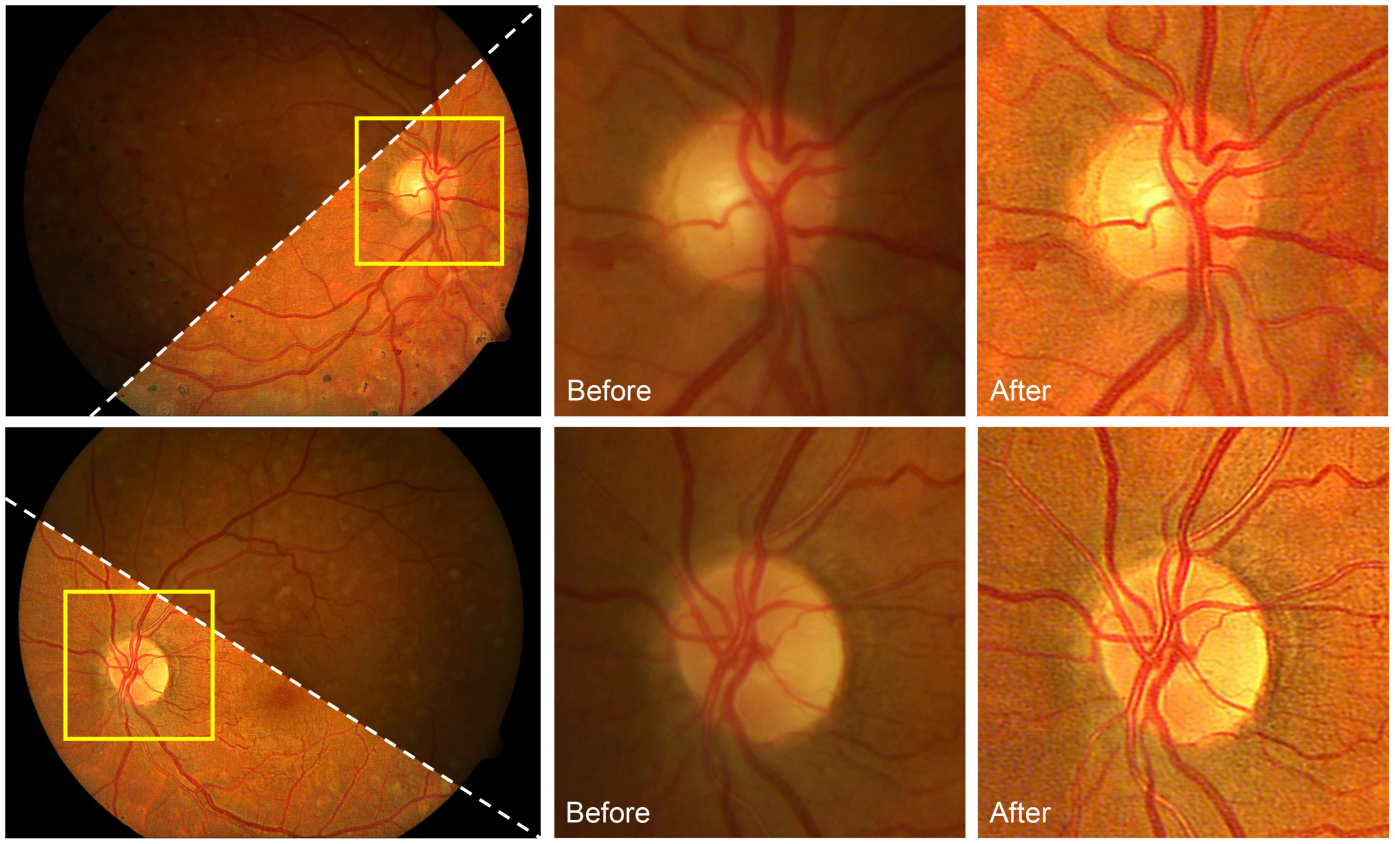
Restoration of retinal image using blind deconvolution.

In (93), an image formation model based on the DPFR feature developed a differentiable non-convex cost function that jointly achieves illumination correction and blind deconvolution. **Figure 12** shows the results of this approach on retinal image deconvolutions, where the uneven illumination and blurriness are corrected. Nevertheless, the method has limitations. First, the model parameters should be manually adjusted, which is a common drawback of non-learning-based blind deconvolution methods. Second, blind deconvolution can be time-consuming as it requires several iterations for solving the latent images, especially for retinal images with large resolutions. Third, the deconvolution will not significantly increase the image contrast as CLAHE does. Further combination of retinal image deconvolution and contrast enhancement is possible to improve the image quality of deconvolution methods.

### 3.4 Deep-learning-based retinal image restoration

With the development of computational power, deep-learning-based retinal image enhancements attracted a lot of interest (94). Because of the lack of paired real retinal images for good and degenerated quality, most learning-based retinal image restoration methods published recently can be categorized as extensions of GAN. These methods convert the retinal image restoration task into a style-transform task that transforms the image style from a bad-quality retinal image to a good-quality one. To mitigate the risk of GANs introducing unexpected artifacts, many focus on preserving information fidelity.

Since there are no paired real retinal images, researchers use synthetic/simulated degenerated retinal images to train the networks. For instance, based on the image formation model proposed by Peli et al. (22), Luo et al. (32) trained an unpaired GAN to achieve cataract retinal image dehazing for mild cataract cases. Li et al. (34) proposed an annotation-free GAN for cataractous retinal image restoration. Based on the natural scene haze formation model, Yang et al. (95) trained a modified cycle-GAN for artifact reduction and structure retention in retinal image enhancement. Shen et al. (96) proposed a new mathematical model to formulate the image-degrading process of fundus imaging and train a network for retinal image restoration. Others have modified the structures of the network or loss function to improve the performance of the networks (97, 98).

**Figure 13:**
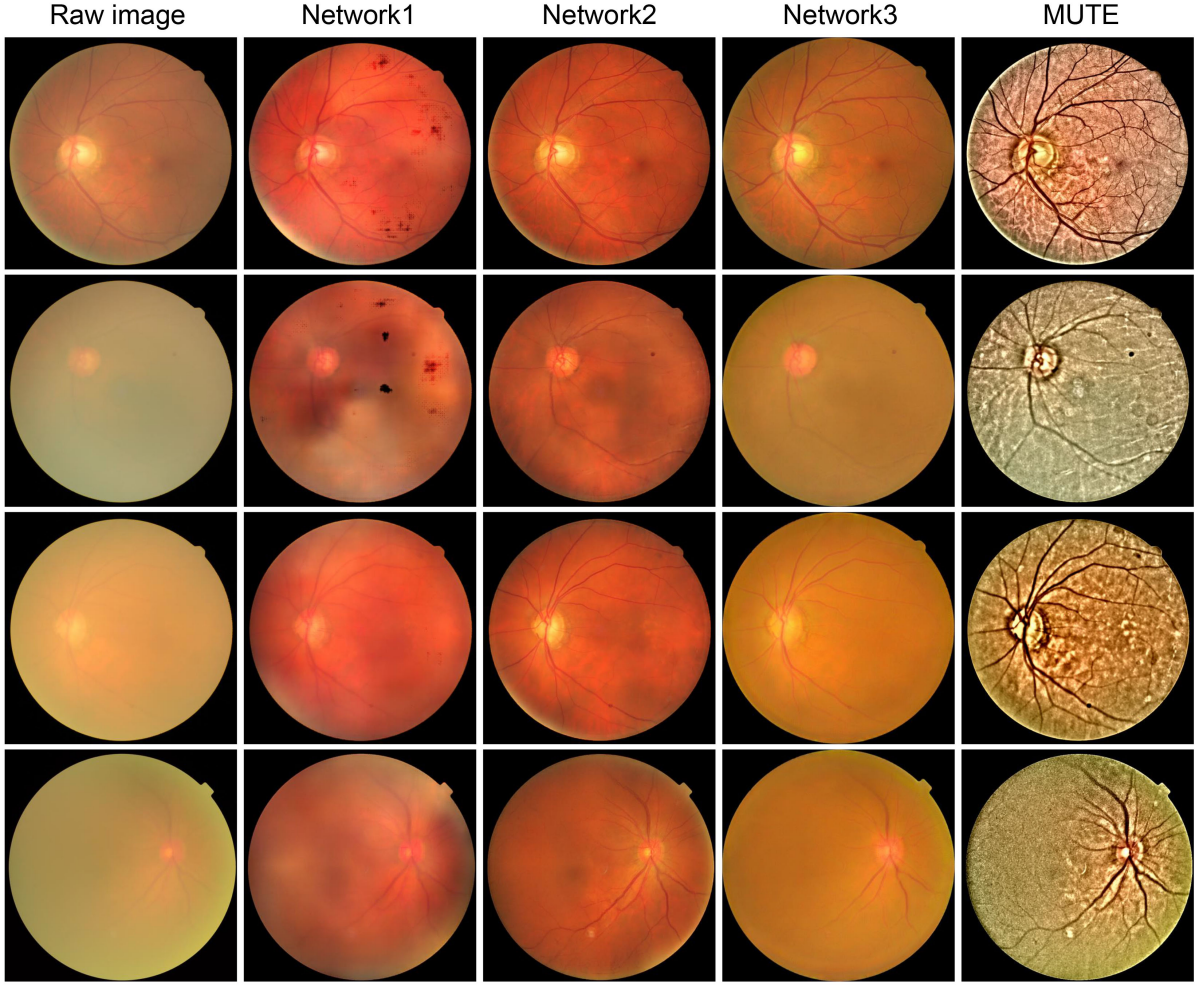
Cataractous retinal image enhancement using three network methods and one nonlearning method. Network 1 (34):. Network 2 (100):. Network 3 (95):. MUTE (101):.

While these learning-based methods produce impressive restoration results in both quality and naturalness preservation, they have limitations. Over-fitting on synthetic data and lack of generalization are potential issues as we will show in the experimental sections. Additionally, the performance of trained networks is limited by the input image resolution (typically 512 × 512), which is too small for clinical applications where image resolution, in general, is larger than 2,000 × 1,000 (99). Furthermore, these methods lack interpretability and may introduce unexpected artifacts or elimination of important retinal structures, as shown in **Figure 13**, which can be detrimental to clinical applications. Thus, there is still a long way to go in both technical and ethical aspects of learning-based retinal image enhancement methods (102).

### 3.5 Retinal image quality metrics

Despite the fact that the enhanced images will be finally evaluated by specialists for supporting clinical applications, evaluating the image quality in an objective way is important for understanding and analyzing the performance of different restoration algorithms. The image quality can be calculated, objectively, using well-designed programs, known as quality metrics, with reference-based and non-reference-based ways. Some metrics have been widely used in the field of image processing, and new metrics are still being developed. This subsection gives a brief introduction to the quality metrics used in retinal image analysis.

**Figure 14:**
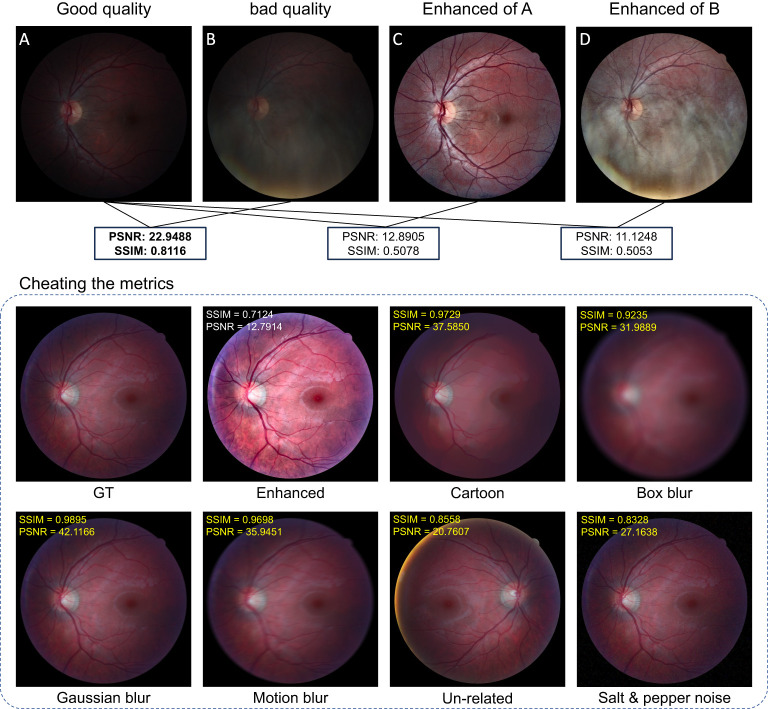
Demonstration of how to cheat the metrics.

The peak signal-to-noise ratio (PSNR) and structured similarity measure (SSIM) are reference-based metrics that can evaluate the image quality if the ground truth is known (73, 103–105). One drawback of these metrics is that they are not consistent with human-visual feeling. Sometimes, they will generate unexpected evaluation results that violate human assessment. To illustrate the problem, we collect good-quality (GQ) images (18 images) and the corresponding bad-quality (BQ) images (18 images) from the HRF dataset (106). We also perform illumination correction and contrast enhancement using CLAHE on both GQ and BQ images. One example is shown in **Figure 14**.

We calculated the PSNR and SSIM between (1) GQ images and BQ images (2), GQ images and enhanced GQ images, and (3) GQ images and enhanced BQ images as listed in **Figure 14**. In all these calculations, the GQ images were set as the reference images as shown in **Figure 14A**, while the metrics imply that **Figures 14C, D** have worse image quality than Figure 14B, which conflicts with human visual assessment. In this case, the decrease in PSNR and SSIM is largely due to the change of the image’s intensity level, especially for SSIM as it depends on the intensity level between the given image and the reference image. Since uneven and insufficient illumination are common problems in fundus images, using PSNR and SSIM to assess the performance of our proposed model lacks fairness and practicality, especially for medical images where the real ground truth is not well-defined. Additional examples that demonstrate the unpredictable behavior of SSIM and PSNR are shown in **Figure 14**. Even the image is degraded by some major types of distortions such as Gaussian blur, motion blur, and noises, and the related SSIM and PSNR scores can be larger than the enhanced image, which far contradicts human visual prospect. More related works showing the drawback of SSIM and PSNR can be found in (73, 107–109).

Reference metrics are usually not applicable since a good reference for medical image usually does not exist or is hard to obtain. As such, non-reference metrics are developed to score the image’s quality based on human visual sensation. The underwater image quality metrics (109) are good candidates to adapt. They include Underwater Image Sharpness Measure (UISM) and Underwater Image Contrast Measure (UIConM). Both the UISM and the UIConM do not rely on the statistical property of images and thus can be applied to retinal images, regardless of the statistical difference between retinal images and underwater images. Moreover, image entropy (IE) describes the randomness distribution of the image and its value denotes the amount of image information (76, 110). The multi-scale contrast of the image, CRAMM, was calculated with a pyramidal multi-resolution representation of luminance (111). Lastly, the fog-aware density evaluator (FADE) (112, 113) was used to numerically predict perceptual hazy density, which can be used to evaluate the image quality of cataractous retinal images.

## 4 Potential applications

### 4.1 Diagnosis

**Figure 15:**
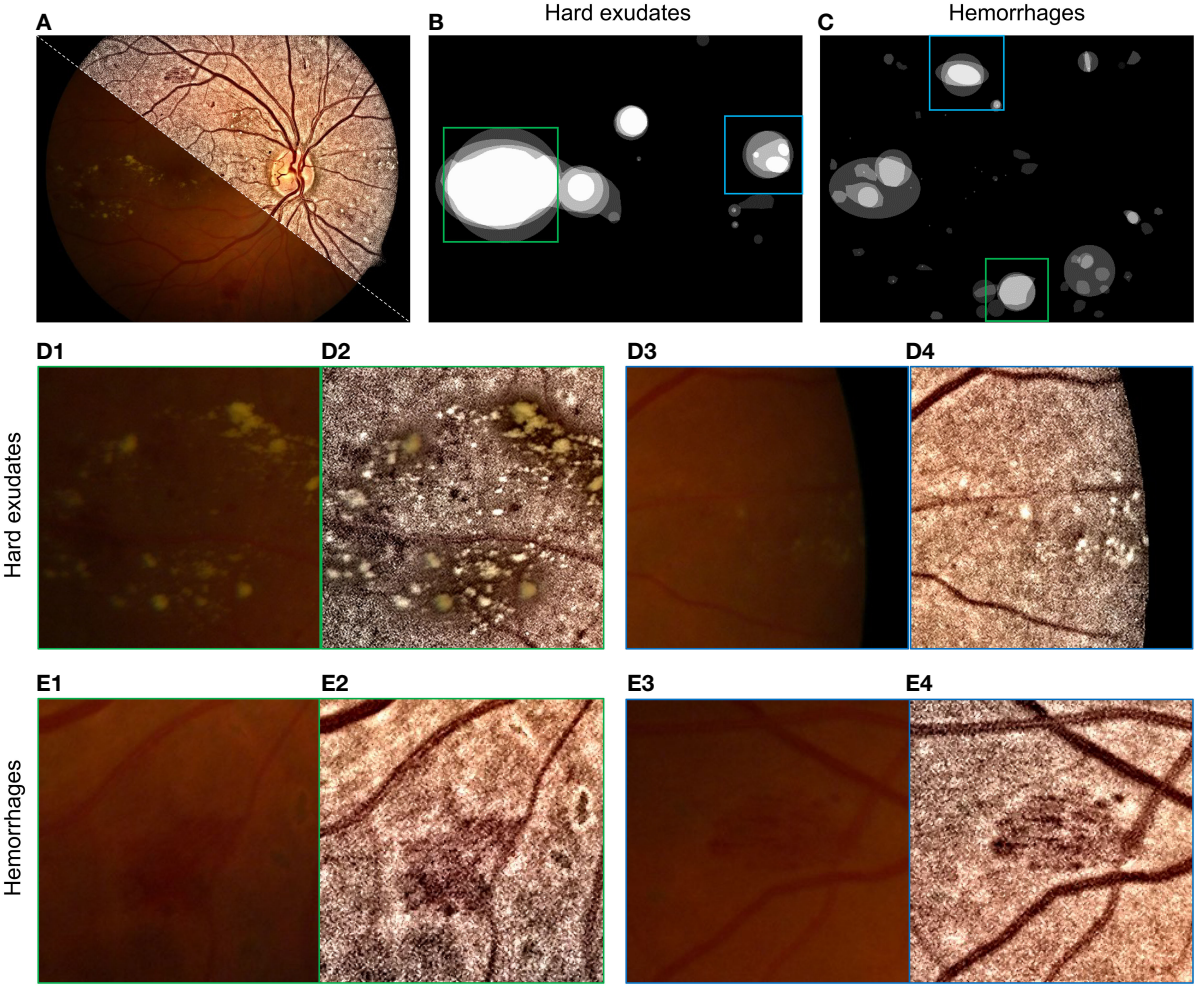
Enhancement of retinopathy areas using (101). **(A)** Montage of raw and enhanced images. **(B)** Labels of hard exudate areas. **(C)** Labels of hemorrhage areas. **(D1–D4)** are enlarged parts of raw and enhanced images corresponding to green and blue boxes in **(B)**. **(E1–E4)** are enlarged parts of **(C)**.

Restored/enhanced retinal images can potentially increase diagnostic accuracy. An example is diabetic retinopathy with areas with hard exudates and hemorrhages, as shown in **Figures 15A, B** and **C**. The enhanced images increased the visual quality of the retinopathy area, without unexpected artifacts to guarantee structure fidelity, as shown in **Figures 15D1–D4**.

Some hard exudates that were barely observable in the raw image (**Figure 15D3**) can be clearly seen in the enhanced images in **Figure 15D4** due to the increased contrast. The enhanced image also has a high visual quality in areas with hemorrhages, as shown in **Figures 1E1–E4**, as the contrast between hemorrhage areas and the background increases in enhanced images (see **Figures 15E2–E4**).

Note that it is of importance to check if algorithms introduce unexpected artifacts or erase the important structure from the image. To do this, one can collect cataractous retinal images before and after cataract surgery, perform the algorithm on the image before cataract surgery, and check if any structures are added or removed by comparing the latter to the actual image after the surgery.

### 4.2 Blood vessel tracking

Retinal image blood vessel segmentation allows parameterization of blood vessels, which is important for clinical diagnosis as morphological changes of blood vessels are biomarkers for diseases such as lacunar stroke (114), cognitive dysfunction (115), cardiovascular risk (116), diabetes (117), and glaucoma (118).

Blood vessel segmentation can be retrieved by either human specialists or computer software. The former provides accurate results but is time-consuming. The latter option provides fast segmentation results but is less accurate compared to human specialists. Moreover, because of the poor image contrast of the cataractous retinal image, hand-based segmentation is even more time-consuming, and automatic segmentation for hazy retinal images can be error-prone. With enhanced retinal image, blood vessel segmentation can be better performed due to the increment of image visual quality (119, 120).

### 4.3 Retinal image registration

Image registration is an important application in computer vision, pattern recognition, and medical image analysis (121–123). It aligns two or more retinal images together to provide an overall comprehensive understanding (121). Retinal image registration relies on precise feature detecting and matching for images to be registered. Registration of cataractous retinal images can be bothersome as the features used to register may be obscured by haze or low-contrast pixels. With the enhancement of image contrast, the registration algorithm can better find the paired feature for accurate registration.

### 4.4 Ultra-wide field retinal image enhancement

Ultra-wide field (UWF) imaging system allows the capture of 200 degrees of the retina (approximately 82% of retinal surface area) in a single shot. It provides non-contact, high-resolution images for clinicians to analyze retinal disorders (124, 125). Imaging using the UWF system can also suffer from illumination problems and haze effects due to imperfect photographing conditions. Here, we demonstrate the application of InQue in enhancing UWF images.

**Figure 16:**
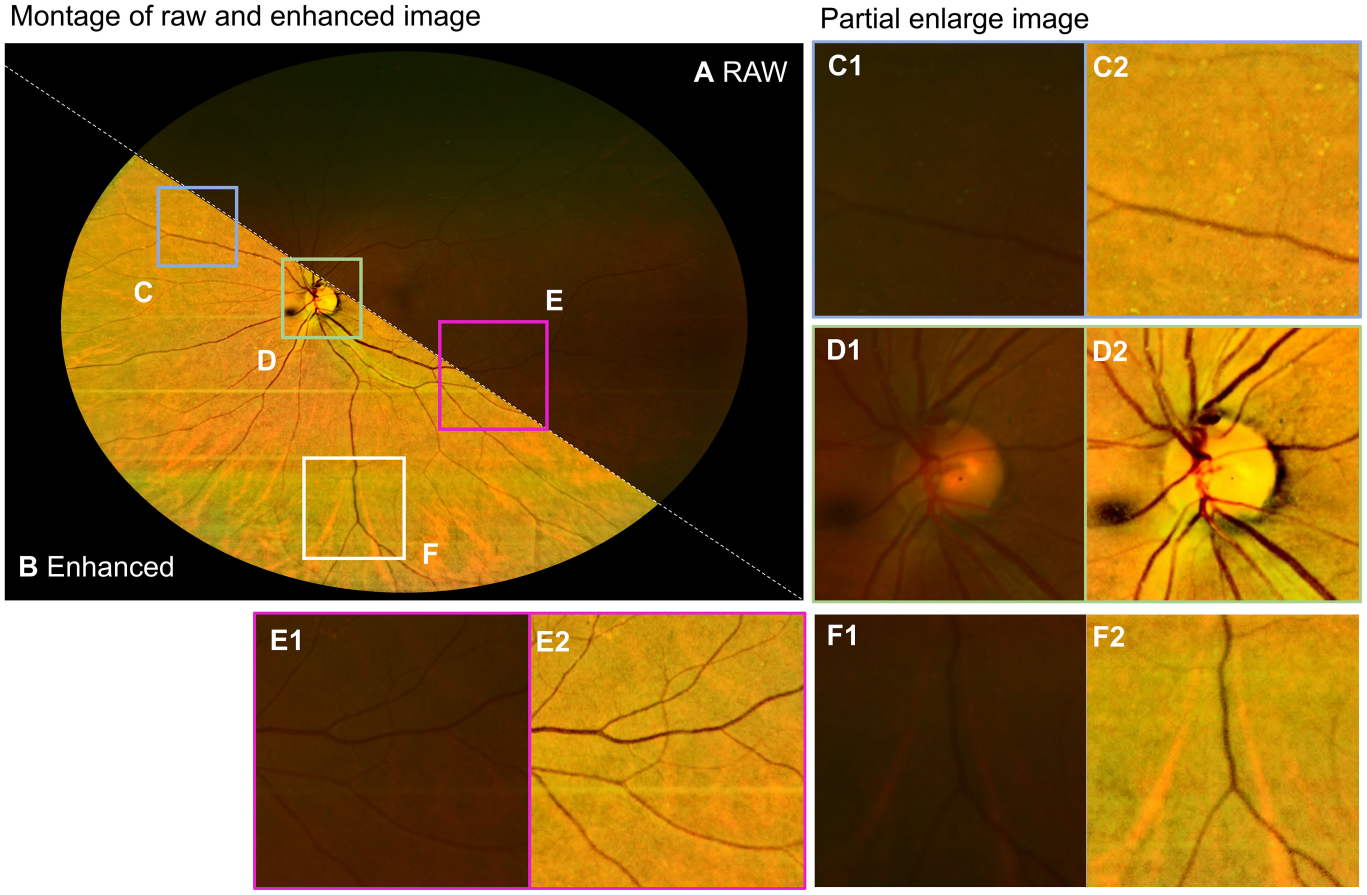
Enhancement for UWF retinal image. **(A)** Raw images. **(B)** Enhanced. **(C1, C2)** are zoomed-in images of the blue box. **(D1, D2)** are zoomed-in images of the green box. **(E1, E2)** are zoomed-in images of the purple box, and **(F1, F2)** are zoomed-in images of the white box.

**Figure 16A** shows the raw image with insufficient illumination and prominent haze. The enhanced one is shown in **Figure 16B**, while the retinal structures including the optical disk and blood vessels are zoomed in **Figures 16C1–F2**. Accordingly, the image clarity of the enhanced image is significantly improved, and blood vessels can be clearly observed.

## 5 Impact of retinal image restoration

### 5.1 Early detection of eye diseases

Retinal image enhancement algorithms improve the quality of images, making it more accurate to identify early signs of eye diseases such as glaucoma, macular degeneration, and diabetic retinopathy (126, 127). Early detection of findings through cataractous retinal images may improve the outcome of treatment of retinal diseases. It may be helpful in the decision-making process of surgery, specifically in combined cases of cataract and retinal disease, to prevent unnecessary interventions (3).

### 5.2 Access to healthcare

The high cost of equipment and the lack of trained professionals can limit access to techniques of retinal imaging, particularly in low-income and rural areas. Low-cost fundus cameras (128, 129) combined with novel image processing algorithms can make retinal imaging accessible and affordable for patients in these areas.

## 6 Conclusion and prospects

We discussed different image formation models and methods of retinal image enhancement/restoration, and how they can be of benefit in clinical applications like blood vessel segmentation, image registration, and diagnosis. These algorithms exhibited variations in their definitions, implementations, underlying structures, and mathematical relationships. Two seemingly unrelated topics, namely, retinal image illumination correction and dehazing, are deeply related to each other in their mathematical insight; thus, a concise description is needed when choosing them for retinal image enhancement.

Ongoing research in retinal image restoration focuses on developing more robust and generalizable methods. This includes addressing challenges related to increasing the performance of non-learning-based methods, which usually distort the naturalness of retinal image after enhancement, negatively affecting clinical applications since some forms of retinopathy are deeply related to the color of the tissues.

## Data availability statement

The original contributions presented in the study are included in the article/supplementary material. Further inquiries can be directed to the corresponding author.

## Author contributions

SZ: Conceptualization, Validation, Methodology, Writing – original draft, Writing – review & editing. CW: Formal analysis, Project administration, Supervision, Validation, Writing – review & editing. TB: Conceptualization, Investigation, Project administration, Supervision, Validation, Writing – review & editing, Formal analysis.
